# Three Airway Management Techniques for Airway Decontamination in Massive Emesis: A Manikin Study

**DOI:** 10.5811/westjem.2019.6.42222

**Published:** 2019-08-06

**Authors:** Michael P. Fiore, Steven L. Marmer, Michael T. Steuerwald, Ryan J. Thompson, Richard E. Galgon

**Affiliations:** * Rosalind Franklin University of Medicine and Science, Chicago Medical School, North Chicago, Illinois; † Des Moines University, College of Osteopathic Medicine, Des Moines, Iowa; ‡ University of Wisconsin School of Medicine and Public Health, Department of Emergency Medicine, Madison, Wisconsin; § University of Wisconsin School of Medicine and Public Health, Department of Anesthesiology, Madison, Wisconsin

## Abstract

**Introduction:**

Emesis occurs during airway management and results in pulmonary aspiration at rates of 0.01% – 0.11% in fasted patients undergoing general anesthesia and 0% – 22% in non-fasted emergency department patients. Suction-assisted laryngoscopy and airway decontamination (SALAD) involves maneuvering a suction catheter into the hypopharynx, while performing laryngoscopy and endotracheal intubation. Intentional esophageal intubation (IEI) involves blindly intubating the esophagus to control emesis before endotracheal intubation. Both are previously described techniques for endotracheal intubation in the setting of massive emesis. This study compares the SALAD and IEI techniques with the traditional approach of ad hoc, rigid suction catheter airway decontamination and endotracheal intubation in the setting of massive simulated emesis.

**Methods:**

Senior anesthesiology and emergency medicine (EM) residents were randomized into three trial arms: the traditional, IEI, or SALAD. Each resident watched an instructional video on the assigned technique, performed the technique on a manikin, and completed the trial simulation with the SALAD simulation manikin. The primary trial outcome was aspirate volume collected in the manikin’s lower airway. Secondary outcomes included successful intubation, intubation attempts, and time to successful intubation. We also collected pre- and post-simulation demographics and confidence questionnaire data.

**Results:**

Thirty-one residents (21 anesthesiology and 10 EM residents) were randomized. Baseline group characteristics were similar. The mean aspirate volumes collected in the lower airway (standard deviation [SD]) in the traditional, IEI, and SALAD arms were 72 (45) milliliters per liter (mL), 100 (45) mL, and 83 (42) mL, respectively (p = 0.392). Intubation success was 100% in all groups. Times (SD) to successful intubation in the traditional, IEI, and SALAD groups were 1.69 (1.31) minutes, 1.74 (1.09) minutes, and 1.74 (0.93) minutes, respectively (p = 0.805). Overall, residents reported increased confidence (1.0 [0.0–1.0]; P = 0.002) and skill (1.0 [0.0–1.0]; P < 0.001) in airway management after completion of the study.

**Conclusion:**

The intubation techniques provided similar performance results in our study, suggesting any one of the three can be employed in the setting of massive emesis; although this conclusion deserves further study. Residents reported increased confidence and skill in airway management following the experience, suggesting use of the manikin provides a learning impact.

## INTRODUCTION

Emesis during airway management is a common event. When it occurs, massive emesis is a major problem as resultant aspiration is associated with both morbidity and mortality.^1^ In the operating room, approximately 0.01% – 0.11% will suffer some complication due to aspiration.^2^ In the emergency department (ED), aspiration rates associated with rapid sequence intubation have been reported from 0% – 22%.^3^ Current guidelines recommend risk assessment and prophylaxis as the basis for aspiration prevention.^4^ Regardless, aspiration events still occur, and operators should be trained in the management of the patient who experiences massive emesis during airway management to mitigate the risk of profound airway contamination and aspiration.

Various techniques and devices have been developed for the management of massive emesis events. One such example is that of a suction laryngoscope studied by Mitterlechner et al.^5^ In that investigation, the authors found a reduction in the number of esophageal intubations (EI) by inexperienced technicians when compared to a standard laryngoscope. However, due to the rarity of emesis events and ethical considerations, supporting evidence is limited. Thus, this is a research area of great need as pulmonary complications, such as pulmonary pneumonitis and pneumonia, lead to patient morbidity. A consensus exists that a pH less than 2.5 and a volume of pulmonary aspirate of greater than 0.3 milliliters per kilogram (mL/kg) is necessary for the development of pulmonary complications.^6^ Patients undergoing emergency airway management who experience massive emesis are likely at risk of meeting these requirements.

Traditionally, the management of massive emesis during intubation includes first positioning the patient in a head-down position, followed by decontamination of the patient’s airway by suctioning, then intubating using either direct laryngoscopy (DL) or video laryngoscopy (VL). Two other techniques have been discussed to manage such an event: suction-assisted laryngoscopy and airway decontamination (SALAD), and intentional esophageal intubation (IEI).^7^–^9^

The SALAD technique, previously described by Ducanto et al.^7^, involves oral airway decontamination while simultaneously preserving VL views for intubation. At the onset of a massive emesis event, the operator clears the airway of vomitus to allow placement of video laryngoscope. With the suction catheter in the right hand and the laryngoscope in the left, the operator advances the suction catheter as a tongue depressor, suctioning vomitus, and allowing for advancement of the laryngoscope. Once a view of the glottis is observed, the operator maneuvers the suction catheter around the laryngoscope blade and uses his or her left hand to hold it in place, thereby freeing the right hand for placement of the endotracheal tube (ETT).

The IEI technique, previously described by Sorour et al.,^9^ involves intentionally intubating the esophagus to achieve control of massive emesis, oral cavity decontamination, and endotracheal intubation. At the onset of a massive emesis event, the operator blindly places the ETT. After the ETT is placed and the cuff is inflated, if the tube is placed in the esophagus the hope is that vomitus is controlled by shunting it away from the patient via the ETT. This then allows for the oropharynx decontamination and endotracheal intubation.

**Population Health Research Capsule**

What do we already know about this issue?

*Emesis during airway management is common, particularly in the emergency department, and can result in serious morbidity and mortality*.

What was the research question?

*This study compared the effectiveness of three intubation techniques using a massive emesis manikin model*.

What was the major finding of the study?

*The intubation techniques performed similarly, suggesting any of the three techniques can be used during massive emesis*.

How does this improve population health?

*This is the first study to investigate intubation technique effectiveness during massive aspiration and will likely spur future research to improve patient care*.

However, no data exists comparing the effectiveness of either of these techniques because studying these techniques in human trials would be particularly challenging and a suitable animal model does not exist. Recently, a traditional airway-training manikin was modified that provides a realistic model of massive emesis or upper gastrointestinal hemorrhage during airway management.^7^,^10^ The SALAD simulation manikin^7^,^10^ consists of a standard airway-training manikin with vinyl tubing attached to the manikin’s esophageal port connected to a self-priming, drill-powered fluid pump. The fluid pump is connected to a container filled with a mixture of aspirate (vinegar and xanthum gum). When the fluid pump is activated, aspirate is pumped into the oral cavity of the manikin. The aspirate flow rate to the manikin is adjustable by the operator. The exact SALAD simulation manikin build used during this trial was similar to the original build described by DuCanto et al. except for the modification of using a 1/10 horsepower submersible utility pump (Ace Hardware, Oak Brook, Il) instead of a drill-driven pump.

The objective of this pilot study was to compare the effectiveness of three different airway management techniques (traditional, IEI, and SALAD) for airway decontamination and tracheal intubation in the setting of a simulated massive emesis with resultant airway contamination. Secondarily, we explored the perceived learning impact of using the SALAD simulation manikin.

## METHODS

This study is a single-center, open-label, randomized controlled trial conducted at the University of Wisconsin Hospitals and Clinics (UWHC). The University of Wisconsin School of Medicine and Public Health (UWSMPH) institutional review board approved this study, and informed written consent was obtained from all study subjects.

**Figure 1:**
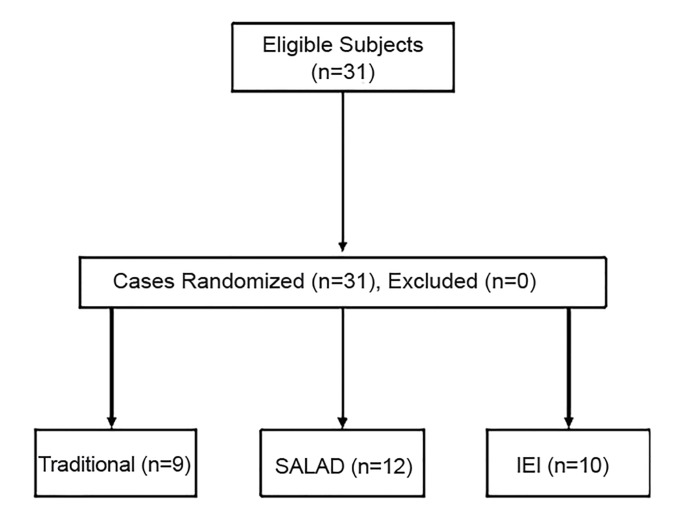
CONSORT diagram. *SALAD*, Suction Assisted Laryngoscopy Airway Decontamination; *IEI*, intentional esophageal intubation.

Senior anesthesiology and emergency medicine (EM) residents, affiliated with the UWSMPH were invited to participate in the trial. Residents were eligible if they were in either the anesthesiology or EM residency programs in their postgraduate year (PGY) 2, 3 or 4 (Figure 1). The EM residency program affiliated with the UWSMPH is a three-year training program. Participants were block randomized on the basis of specialty training (anesthesia or EM) to one of the three trial arms: traditional, IEI, or SALAD using a random number generator. Before beginning the study, each subject completed a pre-simulation questionnaire, which included demographic questions, level of confidence and skill in airway management during massive emesis, experience handling massive emesis during airway management, and prior experience using simulation to learn airway management skills. On study completion, the residents completed a post-simulation questionnaire regarding their level of confidence and skill in airway management during massive emesis, plan to apply their trained technique, perceived usefulness of the training session, and perceived usefulness of the training simulator.

At study onset, each subject watched a five-minute video demonstrating his or her assigned airway decontamination study technique. Subjects then practiced the technique on a manikin of similar make that had not been modified to vomit. Three successful intubations using the assigned technique were required before the subject could proceed to the simulation.

Once the technique familiarization session was complete, the subject was brought to the study manikin and informed that the patient needed to be intubated using the airway decontamination technique they had just practiced. All subjects were provided with GlideScope (Verathon Inc., Bothell, WA) video laryngoscope and a standard Yankauer suction catheter. The simulation began when vomit was visualized in the manikin’s posterior oropharynx. The simulation ended with successful placement of the endotracheal tube as indicated by air movement in the manikin’s lungs.

**Figure 2:**
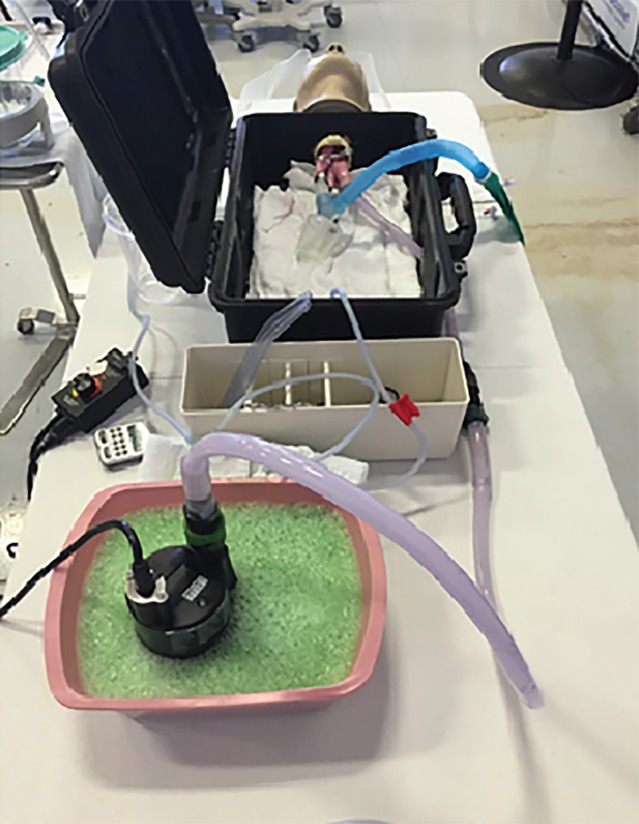
Experimental Setup.

The examiner recorded the time with a stopwatch. A beaker was placed in-line with the right mainstem bronchus of the manikin to collect fluid entering the lungs (Figure 2). The beaker was weighed before and after the examination. The difference was recorded as the volume of aspirate that had entered the lungs. The suction canister was also weighed before and after examination to determine the volume of simulated vomit suctioned by the subject.

The primary study outcome was the compared volume of fluid collected from the lungs between the study arms. To detect a true difference of 25 mL with a variance of 20 mL, a sample size of 12 subjects was required in each arm (alpha = 0.05, beta = 0.80). Secondary study outcomes included successful intubation, time to successful intubation, and the number of intubation attempts for successful intubation. In addition, we collected pre-simulation and post-simulation questionnaire data regarding the training aspects to further investigate the manikin system as a teaching tool. A priori subgroup analysis was performed between PGY status and residents who had vs had not managed the airway of a patient with massive emesis.

We summarized all data by mean (SD), median (IQR), or frequency (%). Demographic and outcome data were compared between randomized treatment methods with analysis of variance or chi-square tests. Post-hoc pairwise comparisons used Holm adjustments to keep a family-wise error rate of 5%. Secondary analyses of outcome measures included grouping subjects by previous experience with patients who have had a massive emesis during airway management, as well as PGY status. These analyses are considered exploratory, and therefore no adjustment for multiple testing over the same outcome measures was done. We conducted similar analyses for the survey questions to assess for differences between groups in confidence and knowledge of management techniques. All tests were conducted at a 0.05 significance level and all analyses were conducted using R version 3.1.1 (Free Software Foundation Inc., Boston, MA).

## RESULTS

After inviting all available anesthesiology and EM residents, 31 residents (21 anesthesiology and 10 EM residents) consented and participated in the study. There were no significant differences in randomization between the three trial arms in terms of age or PGY level (Table 1). The mean (SD) volume of aspirate collected in the lower airway was higher for the IEI and SALAD methods (traditional 72 (45) ml; IEI 100 (45) ml; SALAD 83 (42) ml), but the differences did not reach statistical significance (p = 0.392) (Table 2). Additionally, time to successful intubation was similar between the three groups (traditional 1.69 (1.31) minutes; IEI 1.74 (1.09) minutes; SALAD 1.74 (0.93) minutes; p = 0.805).

Subgroup analysis of residents who had vs had not previously managed massive emesis in a patient during airway management found no difference in mean volume in lungs (82 [48] ml vs 88 [40] ml; p = 0.716) or time to successful intubation (1.71 [1.20] minutes vs 1.74 [0.9] minutes; p = 0.79). PGY-2 residents had higher mean volume in lungs (PGY-2 106 [36] ml; PGY-3 63 [49], PGY-4 91 [38]; p = 0.084) and longer times to successful intubation (PGY2 1.68 [0.71] minutes, PGY-3 1.39 [0.46] minutes, PGY-4 2.10 [1.58] minutes; p = 0.674), when compared to PGY-3 and PGY-4 residents, but these differences did not reach statistical significance (Table 3).

On the pre-simulation questionnaire, PGY-2 residents reported lower confidence ratings (median (IQR)) in managing massive emesis during airway management compared to PGY-3 and PGY-4 residents (PGY-2 3.0 [2.0 – 3.0], PGY-3 3.0 [3.0 – 3.0], PGY-4 3.0 [3.0 – 3.0]; p = 0.046) (Table 4). On a post-simulation questionnaire, residents overall reported a statistically significant increase in confidence ratings in airway management skills and skill in airway suction techniques after completing the study (1.0 [0.0–1.0], p = 0.002; 1.0 [0.0–1.0], p < 0.001). However, PGY-2 and PGY-3 residents thought the training was more useful compared to PGY-4 residents (PGY-2 5.0 [4.0 – 5.0]; PGY-3 5.0 [4.0 – 5.0]; PGY-4 4.0 [3.5 – 4.0]; p = 0.018) and planned on applying the trained technique (PGY-2 4.0 [4.0 – 5.0]; PGY-3 4.0 [4.0 – 5.0]; PGY-4 4.0 [3.5 – 4.0]; p = 0.014).

## DISCUSSION

The main conclusion from our study is that the three intubation techniques provided similar performance results, suggesting any of the three techniques can be employed in the setting of massive emesis. However, the traditional method of intubation during massive emesis, while statistically similar, tended to outperform IEI and SALAD in controlling aspirate volume in the lower airway. To explore the trend, but lack of statistical significance further, given the smaller-than-planned sample size for our study (see Limitations section below), we assessed the effect size of the volume aspirate data by looking at eta squared. The result (0.069) indicates that group designation accounted for 6.9% of the variability in the outcome, which according to Cohen et al.^11^ guidelines, suggests there is at least a medium effect of group designation on lower airway aspirate volume, and that the study’s small sample size is the reason for the non-significant statistical test. Similarly, the results for time to successful intubation followed an analogous pattern (traditional 1.69 minutes [1.31] vs IEI 1.74 minutes [1.09] vs SALAD 1.45 minutes [1.17]; p = 0.805).

The SALAD simulation manikin proved an effective simulator and airway management trainer. The simulator, developed and studied by DuCanto et al., was found to improve the reported overall airway management confidence in a diverse group of learners.^7^ Similarly, we demonstrated that the SALAD simulator manikin is a useful teaching tool. EM and anesthesia residents across different levels of training reported a statistically significant increase in confidence ratings in overall airway management skills and skill in airway suction techniques before and after our simulation (1.0 [0.0–1.0], p = 0.002; 1.0 [0.0–1.0], p < 0.001). Based on our survey results and the study conducted by DuCanto et al., the SALAD simulation manikin has utility as a teaching tool for intubators of all levels in different specialties.

Simulation is fast becoming a popular and effective tool to improve health professional education. Cook et al.^12^ found that in comparison to no intervention, technology-enhanced simulation has had positive effects on “outcomes of knowledge, skills, and behaviors and moderate effects for patient-related outcomes.” Therefore, simulation-based airway management training would likely help health professionals because of the rarity with which emergencies requiring special techniques occur. This idea is supported by Kennedy et al.,^13^ whose group provided evidence that a simulation-based airway management curriculum was more effective in comparison to no simulation interventions, and that simulation was associated with a higher learner satisfaction.

Of note, PGY-4 residents using the SALAD simulation manikin indicated they did not plan on applying the trained technique as much as the junior residents. We suspect that PGY-4s, being farther along in their career, are less impressionable than the PGY-2s and PGY-3s. Additionally, PGY-4s may be more familiar with a specific technique of airway decontamination and less willing to explore new techniques. PGY-2 and PGY-3 residents, still working to build foundational experiences, are more open to developing new airway management techniques. Thus, incorporating training with the manikin earlier in a resident’s career will perhaps have a more significant influence on the development of a resident’s airway management preferences.

## LIMITATIONS

Several limitations existed in our study. The first, and most significant, was the failure to enroll an adequate number of residents to fulfill the power requirement. This resulted from a relative few number of eligible residents at our institution combined with a relative lack of interest. As discussed above, this impacted the statistical test result (ie, a failure to reach statistical significance for observed differences), while our exploration of the effect size suggests at least a medium effect associated with group assignment on the primary study outcome. Therefore, it is reasonable to believe the observed differences in the primary study outcome between the study groups are true, and this deserves further study in a larger investigation.

Second, the simulator itself may have influenced the results of the study. The simulator has a stiff supraglottic area, allowing the residents to easily insert the laryngoscope blade and quickly view the vocal cords. In clinical practice, the resident would likely be more careful and systematically insert the laryngoscope blade. Slower insertion of the laryngoscope blade would allow for more volume to fill the hypopharynx and lungs. Thus, the stiffness of the simulator could have decreased the mean volume of aspirate in lungs and time to successful intubation of the traditional and SALAD techniques.

Next, the consistency of the simulated vomit could have impacted the study. Due to our pumping system, we were unable to provide a “chunkiness” to the simulated vomit that would simulate half-digested, recently-chewed food. These food particles act as obstacles to operators intubating patients and could have provided a realistic challenge in determining the effectiveness of the techniques. For example, IEI could be less hampered by the food items aspirated because there is less reliance on commonly used, rigid suction catheters that are often obstructed by such particles.

Lastly, due to variation in the day-to-day viscosity of the simulated vomit, some residents experienced a slightly different simulation. While a servomotor was used to control flow rate and tests were run to ensure the consistency of the flow rate, slight differences were anecdotally experienced between the trial runs. Had the pump run faster for one of the specific techniques, this could have impacted the performance results.

## CONCLUSION

This is the first study to attempt to assess the efficacy of different methods available for managing massive emesis during airway management. Our findings suggest the three tested methods provide similar results in our simulated model. A larger study with more power or additional operator training in the novel methods (ie, IEI and SALAD) is needed to determine more definitive results. Our study subjects reported the modified airway manikin provides reasonably realistic simulation for managing massive emesis or upper gastrointestinal hemorrhage and may also be useful as a simulator for airway management. Survey results suggest training with the manikin may impart a learning effect.

## Figures and Tables

**Table 1:** Baseline Characteristics.

	IEI (n=10)	SALAD (n=12)	Traditional (n=9)	p-value
Sex, Female	2 (20.0%)	2 (16.7%)	5 (55.6%)	0.169
Resident				0.8
CA	6 (60.0%)	8 (66.7%)	7 (77.8%)	
EM	4 (40.0%)	4 (33.3%)	2 (22.2%)	
Age	31.9 (7.2)	30.2 (2.5)	29.7 (2.3)	0.526
PGY				0.241
2	5 (50.0%)	3 (25.0%)	1 (11.1%)	
3	1 (10.0%)	5 (41.7%)	5 (55.6%)	
4	4 (40.0%)	4 (33.3%)	3 (33.3%)	

Data are mean ± standard deviation or number and percent.

*CA*, clinical anesthesia; *EM*, emergency medicine; *PGY*, post graduate year; *SALAD*, Suction Assisted Laryngoscopy Airway Decontamination; *IEI*, intentional esophageal intubation.

**Table 2:** Primary and Secondary Outcomes of Airway Management Technique.

	IEI (n=10)	SALAD (n=12)	Traditional (n=9)	p-value
Volume in lungs (mL)	100 (45)	83 (42)	72 (45)	0.392
Successful intubation	10 (100.0%)	12 (100.0%)	9 (100.0%)	1
Time to intubate (min)*	1.74 (1.09)	1.74 (0.93)	1.69 (1.31)	0.805
Intubation attempts	1.56 (1.29)	1.45 (1.17)	1.5 (1.28)	0.85

Reported as mean (SD).

* p-value from test based on log transformed data.

*SALAD*, Suction Assisted Laryngoscopy Airway Decontamination; *IEI*, intentional esophageal intubation.

**Table 3:** PGY Subgroup Analysis of Primary and Secondary Outcomes.

	PGY 2 (n=9)	PGY 3 (n=11)	PGY 4 (n=11)	p-value
Volume in lungs (mL)	106 (36)	63 (49)	91 (38)	0.084
Successful intubation	9 (100.0%)	11 (100.0%)	11 (100.0%)	1
Time to intubate (min)*	1.68 (0.71)	1.39 (0.46)	2.10 (1.58)	0.674
Intubation attempts	1.48 (1.21)	1.41 (1.18)	1.5 (1.28)	0.315

Reported as mean (standard deviation).

* p-value from test based on log transformed data.

*PGY*, post graduate year; *mL*, milliliters; *min*, minutes.

**Table 4:** Survey Analysis Based Off PGY Status.

	PGY 2 (n=9)	PGY 3 (n=11)	PGY 4 (n=11)	p-value
Pre-training				
Confidence in airway management	3 (2 – 3)	3 (3 – 3)	3 (3 – 3)	0.046
Skill in airway suction techniques	3 (2 – 3)	3 (2 – 3)	3 (3 – 3)	0.099
Post-training				
Confidence in managing vomiting case	3 (3 – 4)	3 (3 – 4)	4 (3 – 4)	0.387
Skilled with various suction techniques	3 (3 – 4)	3 (3 – 4)	4 (4 – 4)	0.497
Plan to apply trained technique	4 (4 – 5)	4 (4 – 5)	4 (4 – 4)	0.014
Was training useful	5 (4 – 5)	5 (4 – 5)	4 (4 – 4)	0.018
Simulator realistic to challenge skills	4 (4 – 5)	4 (4 – 5)	4 (4 – 4)	0.425

Data are median (interquartile range).

*PGY*, post graduate year.
